# Formation of phage lysis patterns and implications on co-propagation of phages and motile host bacteria

**DOI:** 10.1371/journal.pcbi.1007236

**Published:** 2020-03-13

**Authors:** Xiaochu Li, Floricel Gonzalez, Nathaniel Esteves, Birgit E. Scharf, Jing Chen

**Affiliations:** 1 Department of Biological Sciences, Virginia Polytechnic Institute and State University, Blacksburg, Virginia, United States of America; 2 BIOTRANS Graduate Program, Virginia Polytechnic Institute and State University, Blacksburg, Virginia, United States of America; 3 Fralin Life Sciences Institute, Virginia Polytechnic Institute and State University, Blacksburg, Virginia, United States of America; Institute for Disease Modeling, UNITED STATES

## Abstract

Coexistence of bacteriophages, or phages, and their host bacteria plays an important role in maintaining the microbial communities. In natural environments with limited nutrients, motile bacteria can actively migrate towards locations of richer resources. Although phages are not motile themselves, they can infect motile bacterial hosts and spread in space via the hosts. Therefore, in a migrating microbial community coexistence of bacteria and phages implies their co-propagation in space. Here, we combine an experimental approach and mathematical modeling to explore how phages and their motile host bacteria coexist and co-propagate. When lytic phages encountered motile host bacteria in our experimental set up, a sector-shaped lysis zone formed. Our mathematical model indicates that local nutrient depletion and the resulting inhibition of proliferation and motility of bacteria and phages are the key to formation of the observed lysis pattern. The model further reveals the straight radial boundaries in the lysis pattern as a telltale sign for coexistence and co-propagation of bacteria and phages. Emergence of such a pattern, albeit insensitive to extrinsic factors, requires a balance between intrinsic biological properties of phages and bacteria, which likely results from coevolution of phages and bacteria.

## Author summary

Coexistence of phages and their bacterial hosts is important for maintaining the microbial communities. In a migrating microbial community, coexistence between phages and host bacteria implies that they co-propagate in space. Here we report a novel phage lysis pattern that is indicative of this co-propagation. The corresponding mathematical model we developed highlights a crucial dependence of the lysis pattern and implied phage-bacteria co-propagation on intrinsic properties allowing proliferation and spatial spreading of the microbes. In contrast, extrinsic factors, such as overall nutrient level, do not influence phage-bacteria coexistence and co-propagation. Findings from this work have strong implications for dispersal of phages mediated by motile bacterial communities, which will provide scientific basis for the fast-growing applications of phages.

## Introduction

Viruses that specifically target bacteria, bacteriophages or phages, are critical components of the microbial world. They are found in almost every natural environment, including soil, waters, oceans, and bodies of macroorganisms (e.g., human guts) [1–3]. Furthermore, they are the most abundant organisms in the biosphere [2]. Through their interactions with bacteria, phages constantly regulate the ecology, evolution, and physiology of microbial communities [1, 2]. Because of their antimicrobial activity, the application of phages in food processing, agriculture, and medicine has exploded in recent years [4–6]. Development of these applications benefits from fundamental knowledge about how phages interact with bacteria in a microbial community and how they are dispersed in their microenvironment.

As obligate parasites of bacteria, phages must coexist with their hosts at the population level [1]. This coexistence, however, appears rather inconceivable because phages have a huge proliferative advantage over bacteria. The generation cycles of phage and bacteria fall in comparable time frames, with the phage latent period and bacterial division cycle both on the order of an hour [7]. But in each generation cycle a bacterium produces two daughter cells, while one phage produces ~100 new phage particles. Thus, it would follow that phages would quickly outnumber and annihilate the host bacterial population [8, 9]. However, phages and bacteria have coexisted in natural environments for eons. Recent theoretical and experimental studies demonstrated that the evolutionary arms race could maintain coexistence of phages with host bacteria [10–13]. Coevolution could drive a phenotypic and genotypic diversity in the ability of phages to attack the bacteria and the ability of bacteria to resist the attacks, thereby maintaining the balance between phages and host bacteria [10, 14–16]. However, for a successful evolutionary arms race, phages and bacteria need to coexist at least over the time scale required for the emergence of beneficial mutations [8, 9]. It is therefore critical to understand the population dynamics of phage-bacteria systems and conditions for their coexistence below the evolutionary time scale.

Previous studies on coexistence of phages and bacteria mostly focused on well-mixed, nearly homeostatic systems, such as cultures grown in chemostats [17–23]. Naturally occurring systems of phages and bacteria, however, often do not satisfy the conditions found under these defined laboratory settings. Firstly, natural systems typically do not offer a constant environment. Unlike chemostats, where steady levels of nutrients and waste are maintained, natural systems often experience sporadic deposition and replenishing of resources, and fluctuations in other conditions. Secondly, natural systems usually exhibit spatial heterogeneity to various degrees. The spatial inhomogeneity can significantly impact dynamical coexistence in the phage-bacteria systems [8, 9, 24–26].

A critical spatial process in the phage-bacteria system is the migration of bacteria and phages. Many motile bacteria can migrate towards nutrient-enriched areas via chemotaxis. Phages themselves are not motile, so their dispersal relies on either passive diffusion or transport by their hosts. However, diffusion is inefficient for covering long distances. In addition, diffusion of phage particles is typically reduced by higher bacterial densities and increased viscosities due to bacterial exopolysaccharide production in biofilms [27–29]. Therefore, spatial dispersal of phages mostly relies on infection of and transportation by their motile host bacteria. It is poorly understood how phages and bacteria in a constantly migrating microbial community achieve coexistence, which implies their co-propagation in space.

**Fig 1:**
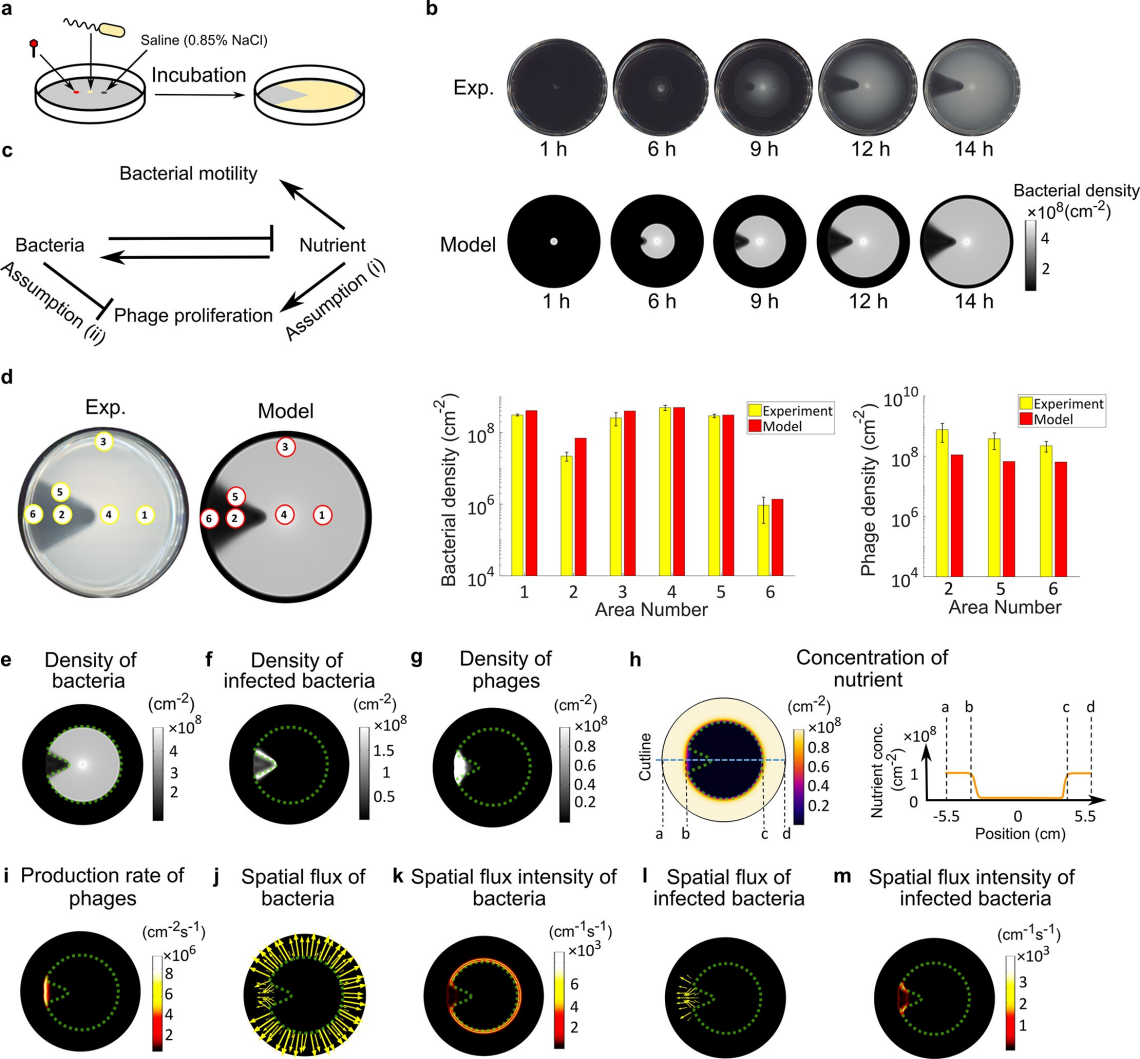
Sector-shaped lysis patterns emerge due to nutrient depletion. (a) Schematic of experimental procedure. Bacteria, phages, and a saline control were spotted on swim plates and incubated for 14 h, which resulted in the development of the sector-shaped lysis pattern. (b) Time evolution of the lysis pattern in experiment vs. model. (c) Interactions between key processes in the model. Pointed arrows: positive influences. Blunt arrows: negative influences. (d) Quantitative comparison of bacterial and phage densities between experiment (yellow bars) and model (red bars). In the experiment, areas labeled by yellow numbers were sampled for phage and bacterial quantifications (see Methods). Corresponding areas in the model are labeled by red numbers. (e) Simulated density of total bacteria. The dashed green outline of the bacteria-dense area is superimposed on (f-m) for reference. (f) Simulated density of infected bacteria. (g) Simulated density of phages. (h) Simulated nutrient concentration (per unit area). Right: Orange curve shows the nutrient concentration profile along the axis of symmetry of the lysis pattern (blue dashed line in left panel). (i) Simulated phage production rate. Active phage production only happens at the outer edge of the lysis area. (j) Simulated spatial flux of total bacteria. (k) Intensity of bacterial spatial flux (~ length of arrow in (j)). Bacteria are motile only at the outer edge of the swim ring. (l) Simulated spatial flux of infected bacteria. (m) Intensity of spatial flux of infected bacteria (~ length of arrow in (l)). Infected bacteria are only motile at the outer edge of the lysis area. The spatial flux shown in (j-m) represents the sum of diffusion flux and chemotaxis flux. Length of the arrow is proportional to magnitude of the spatial flux. (e-m) present snapshots of model simulation at 10 h, an intermediate time at which the colony expansion and pattern formation progress steadily.

In this work we explored the co-propagation of phages and motile bacteria using a simple experimental design, in which phages and bacteria were co-inoculated in a soft agar nutrient medium [30] (Fig 1A). The low agar concentration enabled motile bacteria to swim through the matrix, which, in combination with bacterial growth, resulted in the formation of visible “swim rings” [30]. Inoculation of bacteria and phages in separate locations allowed the experimental setup to mirror realistic scenarios in which expanding bacterial populations encounter phages in a spatial domain. The described experiment generated a highly reproducible sector-shaped lysis pattern. This pattern cannot be explained by any previous mathematical models describing phage plaque formation [31–36], which inevitably produce circular patterns (S1 Fig). Here we constructed a new mathematical model for the spatial dynamics of phages and bacteria, which reproduced the observed lysis pattern and revealed local nutrient depletion as the key to formation of the lysis pattern. Moreover, our model revealed that the sector-shaped lysis pattern with straight radial boundaries requires a balance between intrinsic biological properties of phages and bacteria but does not depend on extrinsic factors. Such a pattern was further shown to be a telltale sign for extended spatial co-propagation of phages and bacteria, implying dependence of co-propagation on intrinsic balance between phages and bacteria. This is the first time that a sector-shaped lysis pattern has been reported in phage-bacteria systems. Our study of this phenomenon via an integrated modeling and experimental approach provides critical insights into naturally occurring dispersal and cohabitation of phages infecting motile bacteria.

## Results

### Nutrient depletion is critical for formation of the lysis pattern

We designed a series of quantitative experiments based on our previously described phage drop assay [37], which allow a spatially propagating bacterial population to encounter phages. *Salmonella enterica* serovar Typhimurium 14028s and χ phage were inoculated 1 cm apart (Fig 1A) on 0.3% agar plate containing bacterial growth medium [30, 38]. As the bacterial population grew, nutrients were consumed. Due to the low agar concentration, bacteria swam through the matrix and followed the self-generated nutrient gradient via chemotaxis, causing spreading of the bacterial population and the appearance of a swim ring. As the bacterial swim ring expanded, it reached the phage inoculation point. The phages then infected the bacteria and generated a lysis area with low bacterial density in the swim ring (Fig 1B). This experiment gave rise to an intriguing sector-shaped lysis pattern (Fig 1B). Most strikingly, as the bacterial swim ring expanded, the radial boundaries of the lysis area stayed unchanged behind the expanding front, resulting in a frozen or immobilized lysis pattern (Fig 1B, S1 Movie). Once the spreading of the swim ring stopped at the plate wall, the lysis pattern persisted for at least 48 hours.

To understand the formation of this lysis pattern, we constructed a mean-field partial differential equation (PDE) model for the phage-bacteria system (Eqs (1) ~ (4)). Like the previous phage plaque models [31–36], our model depicts the basic processes underlying the proliferation and propagation of phages and bacteria. Namely, the bacteria consume nutrients, divide, and move up the nutrient gradient via chemotaxis-directed swimming motility. Once infected by phages, the bacterium is lysed after a latent period, and releases new phage progeny. Note that the run-and-tumble mechanism of bacterial chemotaxis results in a biased random walk of the bacterial cells up the nutrient gradient. The random walk is expressed in the model as the cell diffusion terms and the bias as the cell drift terms (Eqs (1) and (2)). The diffusion of bacteria characterizes the overall effect of active motility and random tumbling of bacteria. Therefore, diffusion and motility of bacteria will be used interchangeably in the rest of this work. In addition, we incorporated the following new assumptions about phage-bacteria interactions in the model, which are critical elements for generating the sector-shaped lysis pattern (S1 Fig).

- Nutrient deficiency inhibits phage replication (Fig 1C). Because phage replication in the host bacteria requires energy, it is likely reduced at low nutrient levels.
- High bacterial density inhibits phage production (Fig 1C). Inhibition of phage attack by quorum sensing signals has been documented in various bacteria, including *E*. *coli* [39], *Vibrio* [40, 41], and *Pseudomonas* [42], and could be a widespread phenomenon. This inhibition stems from reduction of phage receptors (in *E*. *coli* and *Vibrio*) or other mechanisms. High bacterial density, which induces production of the quorum sensing signal, could hence decrease phage production.

Our model reproduces the lysis pattern observed in the phage drop assay (Fig 1B, S1 Movie) and quantitatively matches the bacterial and phage density profiles throughout different areas of the agar plate (Fig 1D). Both experimental and modeling results displayed the highest bacterial density at the inoculation point (area 4), followed by areas outside the lysis sector (areas 1 and 3), along the radial boundaries of the lysis pattern (area 5), in the middle of the lysis pattern (area 2), and the lowest at the outer edge of the lysis sector (area 6). The predicted phage densities also matched the experimental results, i.e., highest in the middle of the lysis sector and lowest at the edge of the swim ring near the wall of the plate (Fig 1D). It should be noted that the lysis area was not entirely void of bacteria. In both experimental and modeling results, a low density of bacteria remained within the lysis area. In the model, nearly all bacteria in this area are infected bacteria (Fig 1E and 1F). In reality, this subpopulation could also include phage-resistant bacteria, which has not been encompassed in our current model.

The model further reveals local nutrient depletion as the key reason for the lysis pattern to immobilize behind the expanding front of the bacterial swim ring. According to the simulation results, as the swim ring expands, nutrients are depleted within the ring (Fig 1H). Nutrient depletion inhibits both phage production (Fig 1I) and bacterial motility (Fig 1J–1M). Note that phages rely on the infection of motile bacteria to propagate in space, because the passive diffusion of phage particles (*D*_*P*_~1 *μm*^2^*h*^−1^) is negligible compared to the “active” diffusion of bacteria resulting from run-and-tumble (*D*_*B*_~10^5^ *μm*^2^*h*^−1^). Therefore, inhibition of bacterial motility, especially motility of the infected bacteria (Fig 1L and 1M), also hinders spatial propagation of phages. Together, the inhibition of phage production and propagation due to local nutrient depletion and reduction of bacterial motility results in immobilization of the lysis pattern at the interior of the bacterial swim ring. The lysis pattern only actively grows at the expanding front of the swim ring, where nutrient supply from the unoccupied periphery can support active phage production and propagation (Fig 1I–1M).

### The lysis pattern reflects radial projection of phage initiation zone

**Fig 2:**
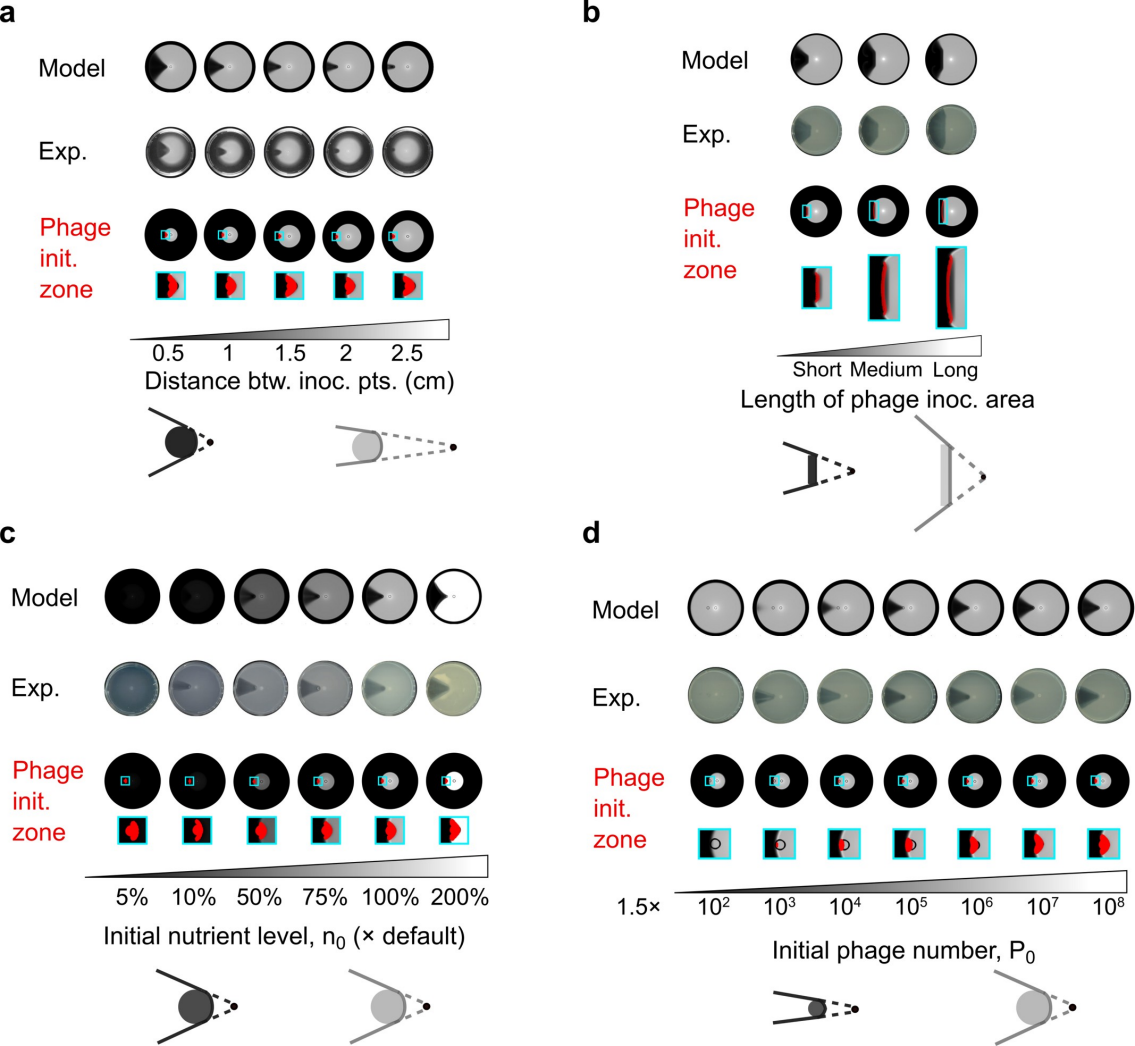
The lysis pattern reflects the radial projection of the phage initiation zone and is insensitive to extrinsic factors. Model and experimental results with (a) phages inoculated at different distances from the bacterial inoculation point, (b) phages inoculated with rods of different lengths, (c) different initial nutrient levels, and (d) different initial phage numbers. In (a-d), the phage initiation zones represent the model-predicted areas occupied by phages when the nutrient level at the center of the phage inoculation area drops below a certain threshold (set at 5% of the initial nutrient level in model simulations). Cartoons summarize scenarios leading to different lysis patterns. Black dots: bacterial inoculation point. Solid dark grey and light grey areas: illustration of phage initiation zones. Solid lines: radial boundaries of lysis patterns. Dashed lines: extension from the bacterial inoculation points to the boundaries of the lysis areas.

Interestingly, the angle of the lysis sector decreased in the experiment when phages were inoculated further away from the bacterial inoculation point and vice versa (Fig 2A). This observation was successfully reproduced and explained by our model (Fig 2A). The lysis patterns in these cases approximately reflect the radial projection from the bacterial inoculation point over an approximately 0.7 cm circle centered at the phage inoculation point (Fig 2A, cartoon). This circle roughly corresponds to the model-predicted area that is occupied by phages when nutrients initially get depleted at the phage inoculation point (Fig 2A, 3rd and 4th rows, nutrient depleted to 5% of initial level). We hereby term this area the “phage initiation zone”, which marks the initialization of the steady expansion of the lysis pattern. Specifically, after the phage initiation zone is established, the phage and bacterial densities at the expanding front remain at a steady level throughout the rest of the pattern formation (S2 Fig).

Our model further predicts how the phage initiation zone and the projected lysis pattern rely on additional factors, which have all been confirmed by experiments (Fig 2). Firstly, the phage initiation zone is predicted to encompass the original phage inoculation area (Fig 2A and 2B). In the corresponding experiments, when phages were inoculated with rods that were significantly longer than the size of the phage initiation zone during point inoculation, the phage initiation zone became dominated by the rod size, and the lysis pattern roughly reflected the radial projection of the phage inoculation area (Fig 2B). Secondly, the size of the phage initiation zone is predicted to remain roughly the same and result in similar angles of the lysis sector, despite changes in total nutrient concentration (Fig 2C). This prediction was also validated by experiment (Fig 2C). Thirdly, the phage initiation zone is predicted to enlarge and result in larger angles in the lysis sector, as the initial phage particle number increases; again, this was validated by experiment (Fig 2D). This result can be understood in that lowering the initial phage number reduces the number of phages being produced by the time nutrients get depleted at the phage inoculation point and shrinks the phage initiation zone (Fig 2D). When the initial phage number is too low, phages fail to establish the initiation zone and subsequently the lysis sector (Fig 2D, *P*_0_ = 1.5×10^2^). The model also predicts that the initial bacteria number does not affect the lysis pattern (S3 Fig). Overall, our modeling and experimental results show that the lysis pattern maintains straight radial boundaries (Fig 2) despite changes in the extrinsic factors tested above, i.e., distance between inoculation point, size of inoculation area, overall nutrient level, and initial phage/bacteria number.

### Competition between phages and bacteria determines shape of lysis pattern

**Fig 3:**
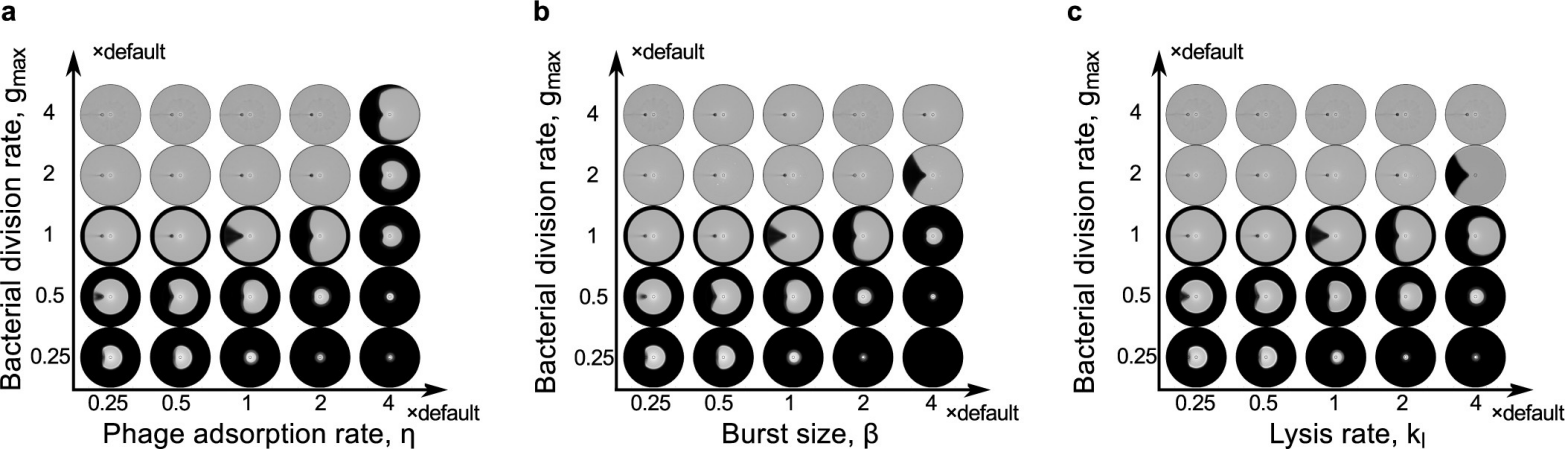
Competition between bacterial and phage proliferation determines the shape of lysis patterns. Simulated lysis patterns with various bacterial growth rate constants versus (a) phage adsorption rate constants, (b) phage burst sizes, and (c) phage-induced lysis rate constants.

Although the straight radial boundaries of the lysis pattern are maintained under various external conditions like nutrient level and initial inoculation, our model predicts a significant change in the lysis pattern when intrinsic biological parameters are altered. In the simulation results, promoting the proliferative efficiency of phages (by increasing phage adsorption rate, phage burst size or lysis rate of infected bacteria) causes the lysis pattern to flare out, and decreasing phage proliferation causes the lysis pattern to close up (Fig 3, horizontal axes). Meanwhile, promoting bacterial proliferation causes the lysis pattern to curve inward and close up, and vice versa (Fig 3, vertical axes). To understand this result, note that both bacterial and phage proliferation depend on nutrient (expressed as increasing functions of local nutrient concentration in the model, see Materials and Methods). As previously shown, proliferation of bacteria causes nutrient to be depleted inside the bacterial swim ring (Fig 1H). Therefore, the bacterial proliferation rate at the expanding front determines how fast nutrients get depleted locally. This time further determines angular spreading of phages along the expanding front of the swim ring, because phage proliferation only thrives before local nutrient depletion. Therefore, either stronger phage proliferation or a weaker bacterial proliferation (causing slower nutrient consumption) allows phages to spread in an accelerated fashion as the swim ring expands, resulting in a flared-out lysis pattern. Vice versa, a weaker phage proliferation relative to bacterial proliferation results in a lysis pattern with edges closing inwards. The sensitivity of the predicted lysis pattern to phage and bacterial proliferation suggests that the experimentally observed lysis pattern requires a balance between the proliferation of the bacterial and phage strains tested.

### Bacterial motility and chemotaxis affect the lysis pattern

**Fig 4:**
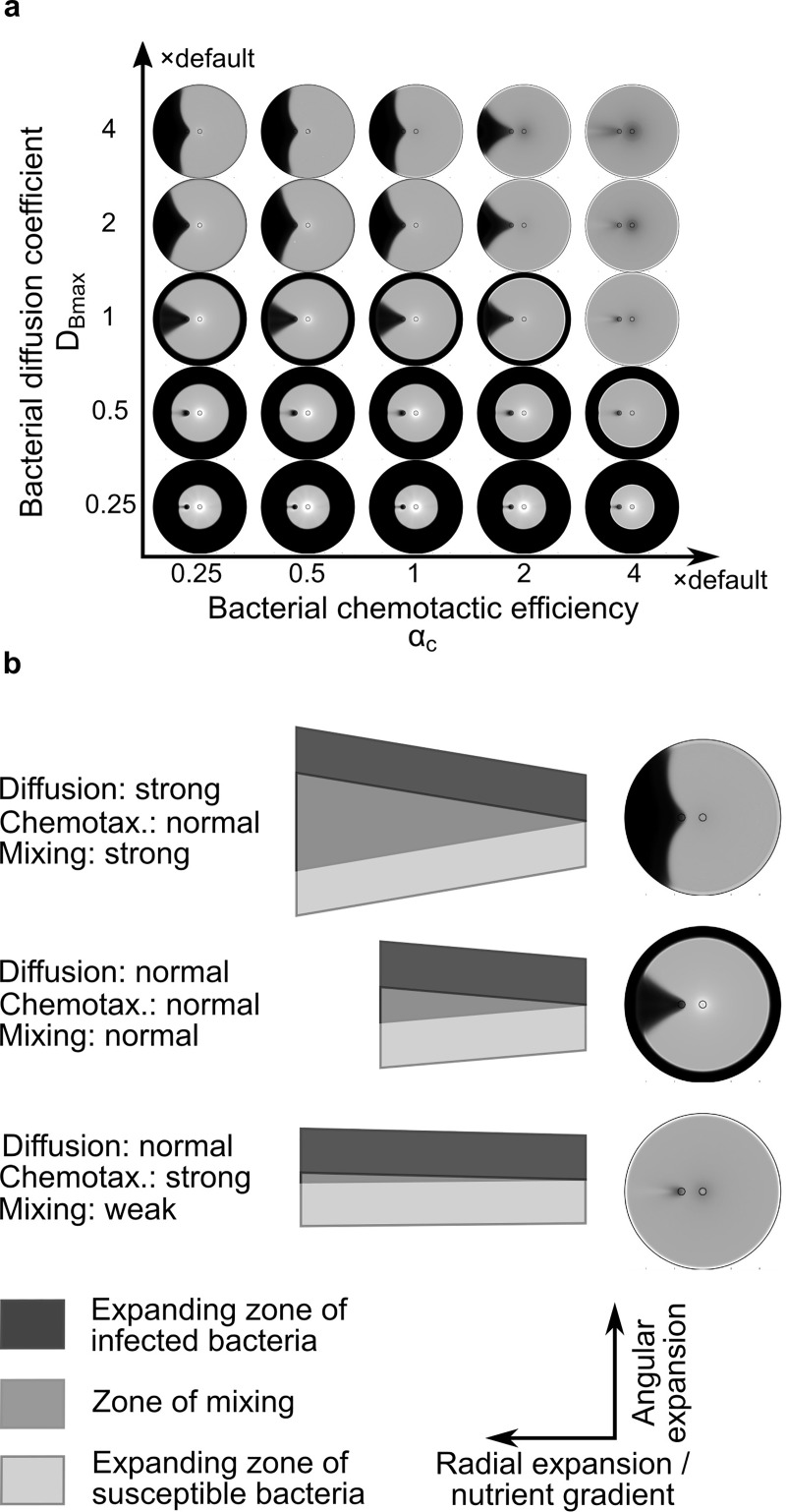
Effects of bacterial motility and chemotaxis on the shape of lysis patterns. (a) Simulated lysis patterns with various bacterial diffusion coefficients and chemotactic efficiencies. The diffusion coefficient in the model reflects the efficiency of bacterial motility. (b) Illustration of how bacterial diffusion and chemotaxis affect the evolution of lysis patterns. The cartoon illustrates a hypothetical history of expansion and mixing of two bacterial patches that would occur along the expanding front of the swim ring. The dark and light grey patches start with infected and susceptible bacteria, respectively. Because nutrient is depleted behind the expanding front, bacterial expansion along the radial axis only occurs in the outward direction. Strong bacterial diffusion promotes expansion equally in all directions, which enhances mixing between the infected and susceptible bacteria and leads to more effective phage propagation and flare-out of the lysis pattern (top row). In contrast, high chemotactic efficiency promotes expansion only against the nutrient gradient in the radial direction, which effectively parallelizes bacterial motion, reduces their mixing in the angular direction, and causes the lysis pattern to close up (bottom row).

We next used the model to investigate how bacterial motility and chemotaxis influence the shape of the lysis pattern. Bacterial motility is reflected by the bacterial diffusion coefficient in the model. A higher cell speed corresponds to a larger diffusion coefficient [43]. Expectedly, a larger diffusion coefficient causes faster expansion of the bacterial swim ring in the model (Fig 4A). The chemotactic efficiency, on the other hand, characterizes the bias of bacterial diffusion. Chemotaxis promotes the directed motility of bacteria in the radial direction due to the nutrient gradient formed by bacterial nutrient consumption (Fig 1H). Consistently, the model predicts that higher chemotactic efficiency expedites expansion of the bacterial swim ring (Fig 4A), because the moving cells at the expanding front can follow the nutrient gradient more efficiently.

The effects of bacterial motility and chemotactic efficiency on the lysis pattern, however, are predicted to be exactly opposite to each other. According to the simulation results, increasing the bacterial diffusion coefficient or decreasing the chemotactic efficiency causes the pattern to flare out (Fig 4A). Vice versa, decreasing the bacterial diffusion coefficient or increasing chemotactic efficiency causes the pattern to close up (Fig 4A). The model generated this result because increasing bacterial diffusion coefficient, i.e., increasing bacterial motility, promotes mixing of infected and susceptible bacteria (Fig 4B, top row). Such mixing is critical for spatial propagation of phages and angular expansion of the lysis pattern, because phages cannot move on their own and rely on infected bacteria to spread in space. In contrast, enhancing chemotaxis inhibits such mixing, because it effectively promotes parallel motion of the bacteria along the radial direction towards the high-nutrient area outside the swim ring (Fig 4B, bottom row). Taken together, bacterial motility and chemotaxis need to be in balance to generate a lysis pattern with straight radial boundaries. Collectively, these findings and those from the model in the previous section indicate that bacterial motility and chemotaxis are required to be in balance with bacterial and phage proliferation rate to generate straight radial boundaries in the lysis pattern (S4 Fig).

To test these model predictions, we performed the phage drop assay with strains of *S*. Typhimurium 14028s containing deletions in two chemoreceptor encoding genes, *tar* and *tsr*. Strains with deletions in *tar* or *tsr* did not significantly change the lysis pattern, whereas the strain containing a deletion of both genes produced a moderate flare-out of the lysis pattern (S5 Fig). This result is qualitatively consistent with the model prediction that weaker chemotactic efficiency causes a pattern with a wider angle (Fig 4A).

We further tuned bacterial motility through varying the agar density. We only experimented with lower agar densities, because higher agar densities are known to switch the mode of bacterial motility to surface swarming, and the results would not be comparable to those obtained from soft agar. In our experiments, softer agar indeed increased bacterial motility, as the bacterial swim ring took a shorter time to reach the edge of the plate (7 h in 0.2%, 9 h 0.25% agar and 14 h in 0.3% agar). However, we found similar sector-shaped lysis patterns regardless of the agar density (S6A Fig). To understand why higher bacterial motility in softer agar did not change the lysis pattern, it is important to note that lowering the agar density also increases the passive diffusion of small molecules such as nutrients [44, 45]. In our model, when bacterial and nutrient diffusion coefficients are proportionally varied, the sector-shaped lysis pattern is indeed maintained (S6B Fig, diagonal from bottom left to top right). The subtle increase of the angle in the pattern in softer agar was also reproduced by the model (S6B Fig, diagonal from bottom left to top right). It is worth noting that agar density *per se* is an extrinsic factor. Although agar density affects bacterial motility, an intrinsic property, the effect of the latter on the lysis pattern is canceled by the accompanying changes in nutrient diffusion. These experimental and modeling results further confirm our previous conclusion that the lysis pattern is insensitive to extrinsic factors.

### Straight radial boundary of lysis pattern is a telltale sign for extended co-propagation

**Fig 5:**
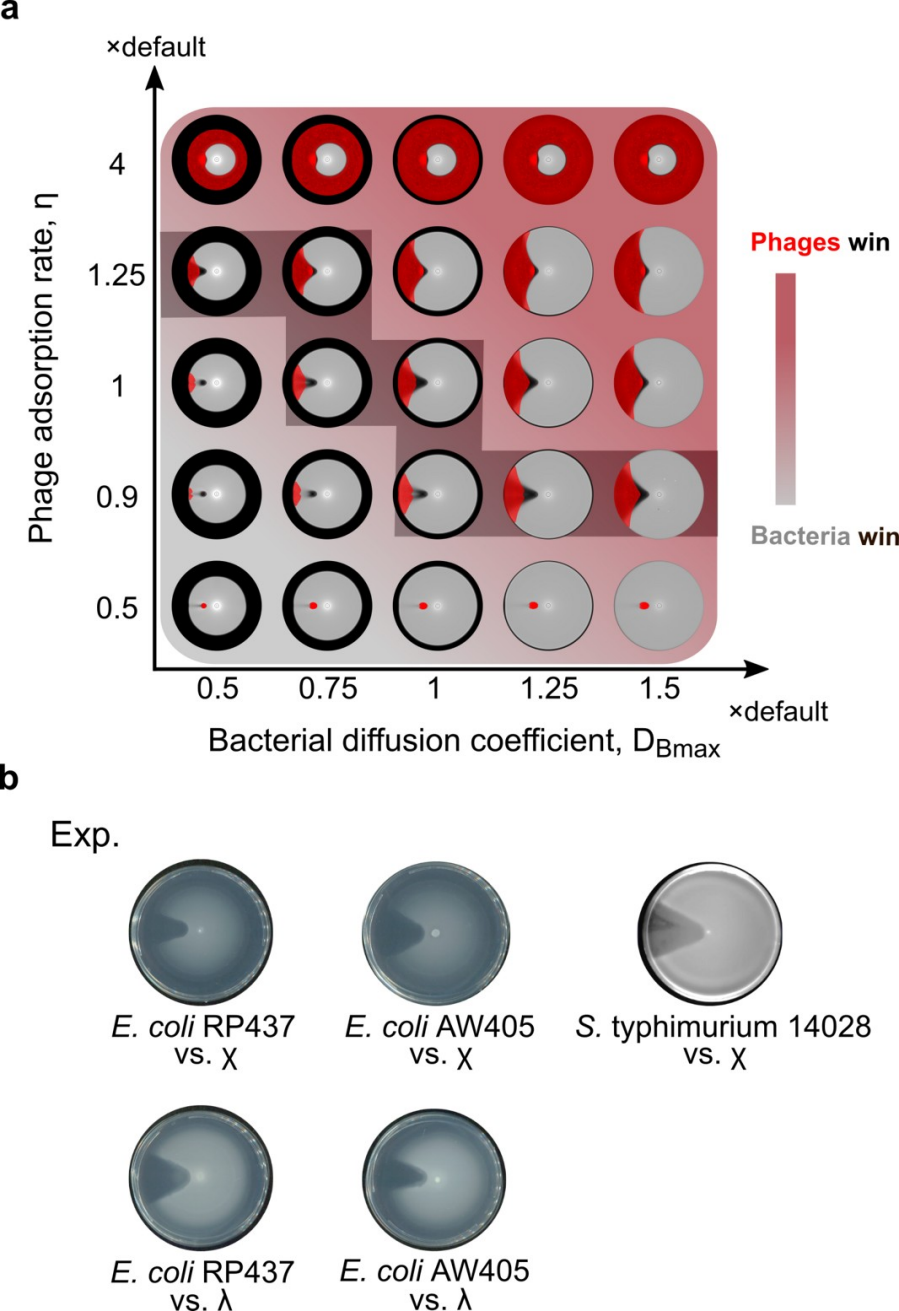
Co-propagation between phages and bacteria is reflected by the straight radial boundaries of the lysis pattern. (a) Lysis patterns and corresponding spatial patterns of phages with various bacterial diffusion coefficients and phage adsorption rate constants. Red/grey gradient in the background illustrates the relative rate at which phages encircle bacteria (red) or bacteria encircle phages (grey). Red shade in simulated patterns: density of phages. Grey shade in simulated patterns: density of bacteria. Grey shadowed staircase: potential trajectory of evolutionary arms race on which phages and bacteria maintain balance with each other. Refer to S7 Fig for clearer, separate view of the lysis patterns and phage patterns. (b) Experimental results from pairs of different bacterial species and their cognate phages.

A closer look at the model results reveals that the straight radial boundaries in the lysis pattern imply co-propagation of bacteria and phages over extended periods (Fig 5). Unlike the sector-shaped pattern with straight radial boundaries, a flared-out or closed-up lysis pattern indicates that one species would outcompete the other during the co-propagation (Fig 5A). For example, the result at the upper right corner of Fig 5A shows a case where phages encircle bacteria and block their further propagation in space. Vice versa, the result at the lower left corner of Fig 5A shows the opposite case where phage propagation is blocked by bacteria. For the less extreme flared-out or closed-up lysis patterns (e.g., Fig 5A, the last column on the 2nd row), one species would eventually encircle and block the other, if the simulation had been run on a larger spatial domain that allow further spatial expansion.

Remarkably, similar sector-shaped lysis patterns with straight radial boundaries were observed in different bacteria-phage pairs (Fig 5B). First, we performed the phage drop assay using *E*. *coli* and phage χ and found a similar sector-shaped lysis pattern (Fig 5B, first row). Since χ is a flagellotropic phage that targets the flagella of the host bacteria [46–48], we further examined whether infection of motile *E*. *coli* by a non-flagellotropic phage, λ, would generate a similar lysis pattern. Phage λ was chosen because of an overlapping bacterial host range with χ. Interestingly, similar sector-shaped lysis patterns were observed independent of the utilized phage type (Fig 5B). Combined with the model findings above and earlier results on sensitivity of the pattern to intrinsic parameters, these highly similar sector-shaped lysis patterns indicated that both *Salmonella* and *E*. *coli* are in intrinsic biological balance with both phages, λ and χ.

## Discussion

In this work, we combined experiments and modeling of lysis pattern formation to investigate the coexistence and co-propagation of phages and bacteria in space. Our experimental setting has strong implications for realistic scenarios, where an expanding bacterial population encounters phages and mediates their dispersal. We observed the formation of an asymmetric, sector-shaped lysis pattern, which cannot be explained by the previous models for lysis pattern formation in phage-bacteria systems. Our new mathematical model successfully reproduced the experimental observation and revealed the importance of nutrient depletion in maintaining the geometric asymmetry initialized in the system. Specifically, local nutrient depletion inhibits phage production and propagation behind the expanding front of the bacterial swim ring, thus immobilizing the lysis pattern. Without the immobilization effect, the lysis pattern would lose asymmetry and eventually reduce to a circle, as predicted by the previous models for phage plaque formation [31–36].

Most importantly, straight radial boundaries of the lysis pattern present a telltale sign that a phage-bacteria system is capable of co-propagation over extended period (Fig 5A). Therefore, the shape of the lysis pattern can serve as a reporter of co-propagation. The model further demonstrated that a straightly expanding pattern requires balance among bacterial proliferation efficiency, phage proliferation efficiency, bacterial motility and bacterial chemotaxis (Figs 3 and 4 and S4 Fig). The balance of biological properties keeps angular expansion of the lysis pattern in pace with its radial expansion and creates straight radial boundaries in the evolving lysis pattern. In contrast, the straight boundary is insensitive to extrinsic factors, including nutrient levels, initial phage/bacterial numbers, agar density, and temperature (Fig 2, S3, S6, S8 and S9 Figs). Together, these findings suggest that balance of intrinsic factors supports robust co-propagation of phages and bacteria regardless of variations in the environment or initial conditions. Interestingly, we experimentally discovered similar sectorial lysis patterns with straight radial boundaries in two different enteric bacterial species paired with two different phages (Fig 5B). These lysis patterns implied intrinsic biological balance between the phages and their bacterial hosts. This phenomenon suggests that natural pairs of bacteria and phages could have shaped their biological properties to allow robust spatial coexistence and co-propagation, likely as a result of coevolution. In the future, we will perform the phage drop assay on other phage-bacteria pairs to examine whether this conclusion may be generalized universally.

The model predictions on extrinsic factors were verified by our experiments (Fig 2, S6 Fig). It was much more complicated, though, to vary the intrinsic biological properties in a controlled fashion. We are relegating these experimental testing to future work, which will also provide feedback for model refinement. For example, the predicted lysis pattern only changed significantly in the model when chemotactic efficiency was increased from the default value (Fig 4A), whereas a flared-out lysis pattern occurred experimentally in a strain lacking two major chemoreceptors (S5 Fig). This quantitative discrepancy indicates that the chemotaxis term and/or parameters in the model should be modified in the future.

Our study specifically underscores that co-propagation of phages and bacteria, i.e., their coexistence in the context of a migrating microbial community, requires not only a balance between the proliferative efficiency of phages and bacteria, but also between their ability to spread in space (autonomous spreading of bacteria vs. bacteria-mediated spreading of phages). This finding brings about an interesting generalized view about what phages and bacteria strive to balance during their coevolutionary arms race. The requirement of balanced proliferative efficiency is known to create the selective pressure that drives the evolutionary arms race between phages and bacteria in their ability to attack and resist attack [10, 12, 14–16]. In light of our finding, the requirement of balanced ability to spread in space could also create a selective pressure to drive an arms race in evolving stronger ability to spread in space. For example, the emergence of flagellotropic phages, i.e., phages specifically targeting actively rotating bacterial flagella [49], could reflect an evolved strategy for phages to improve their ability to propagate in space. Notably, our model predicts a significant impact of chemotactic efficiency on co-propagation of phages and bacteria (Fig 4 and S9D Fig). Therefore, bacteria could, in principle, evolve higher chemotactic efficiency as a counterattack on phage infection. To test this hypothesis, in the future we will examine whether the arms race between phages and bacteria indeed affect the diversity in genes regulating bacterial chemotaxis. Overall, our finding suggests that the coevolutionary arms race (Fig 5A, dark shaded staircase) likely happens in a high-dimensional space that include all properties related to bacterial proliferation and spreading vs. phage proliferation and spreading.

The sector-shaped lysis pattern in our phage-bacteria system is reminiscent of the sector-shaped bacterial patterns formed in range expansion experiments with two bacterial strains [50–53]. Similar to our system, many range expansion experiments demonstrated the important role of spatial factors in coexistence of interacting species in expanding microbial communities, e.g., [50, 51]. However, the underlying process driving pattern formation in the bacterial range expansion experiments is different from that in our phage-bacteria system. In the range expansion experiments, the bacterial patterns result from random genetic drift in the otherwise co-inoculated, well-mixed populations at the expanding front of the bacterial population. The stochastic nature of the genetic drift process causes random fluctuations in the number, size and boundary shape of the patterns. In contrast, the lysis patterns in our system rely not on random genetic shift, but on establishment of the phage initiation zone (Fig 2) as the bacterial population migrates past the phage inoculation loci. The phage initiation zone is established upon a large number of phage particles, with diminishing stochasticity. Hence, our lysis patterns are smooth. Likely for a similar reason, a smooth pattern was observed in a range expansion experiment of two bacterial strains inoculated with a distance apart [54]. Interestingly, although the two engineered bacterial strains in [54] play the roles of predator and prey, respectively, representing similar inter-species interactions as in our phage-bacteria system, the pattern did not exhibit straight radial boundaries and the predator strain won eventually. This difference highlights the capability of extended co-propagation as an evolved property of systems of naturally coexisting species.

Our current work exploited the simplest possible experimental and model setup to understand how phages and bacteria coexist and co-propagate in space, using lytic phages and uniform initial nutrient concentration. In the future, we will modify our experimental and model parameters to investigate additional factors, such as lysogeny and non-uniform nutrient distribution, on the spatial ecodynamics of phage and bacteria. We will also incorporate coevolution between phages and bacteria into our model and experimental set up, to investigate the long-term co-propagation under the effect of evolution. Findings from this work have strong implications for dispersal of phages in microbial communities and lay the groundwork for future applications, such as phage therapy. In the future, we hope to create a model that will aid successful selection and engineering of phages for targeted applications by providing information on phage dispersal and interaction with host bacteria in the corresponding environment. Last but not least, the principles revealed in this work about co-propagation of motile hosts and passive pathogens could be broadly applicable to general host-pathogen communities.

## Materials and methods

### Bacterial strains and phages

The strains of bacteria and phages used are listed in Table 1.

**Table 1:** Biological materials used in this study.

Species/strains/ plasmids	Relevant characteristics	Sources	References
*Salmonella enterica* serovar Typhimurium			
14028s	Wild type	Gift from Rasika M. Harshey	
14028s Tar^-^	*tar*^*-*^	This work	
14028s Tsr^-^	*tsr*^*-*^	This work	
RH2312/SM20	*tar*^*-*^*tsr*^*-*^	Gift from Rasika M. Harshey	
TH2788	*fliY*5221::Tn*10d*Tc (−86 from ATG of *fliY*)	Gift from Kelly T. Hughes	
*Escherichia coli*			
RP437	Wild type	Gift from Howard C. Berg	[55]
AW405	Wild type	Gift from Howard C. Berg	[56]
Phages			
λ phage	*vir*	Gift from Rüdiger Schmitt	[57]
χ phage		Gift from Kelly T. Hughes	
Plasmid			
pKD46	*bla* P_BAD_ *gam bet exo* pSC101 *oriTS*	Gift from Howard C. Berg	[58]

### Media and growth conditions

*Salmonella enterica* serovar Typhimurium 14028s was grown in MSB at 37°C. MSB is a modified LB medium (1% tryptone, 0.5% yeast extract, and 0.5% NaCl) supplemented with 2 mM MgSO_4_ and 2 mM CaCl_2_. *Escherichia coli* strains were grown at 30°C in T-broth containing 1% tryptone and 0.5% NaCl at 30°C.

### Construction of mutant strains

The protocol for lambda-Red genetic engineering [58, 59] was followed to make *S*. Typhimurium mutant lacking *tar*.

### Phage drop assay

Swim plates containing MSB medium (for *S*. Typhimurium) or T-broth (for *E*. *coli*) and 0.3% bacto agar were inoculated with 2.5 μl of a stationary phase bacterial culture in the center of the plate along with 2.5 μl of phage suspension (MOI = 25.4) at a 1 cm distance from the inoculation point. A 2.5 μl spot of 0.85% saline was placed at the same distance from the bacterial inoculation point, opposite from the phage suspension, as a control. Plates were incubated at 37°C (*S*. Typhimurium) or 30°C (*E*. *coli*) for 14 hours. All plates were imaged using the Epson Perfection V370 scanner. Phage drop assays with slight modifications were conducted to test different variables. For the rod-shaped inoculations, sealed glass capillaries of different lengths were immersed in phage suspension and pressed against the soft agar at a distance of 0.5 cm from the bacterial inoculation point. To test the effect of varying inoculation distances, phage suspensions were inoculated at 0.5, 1.0, 1.5, 2.0, and 2.5 cm from the bacterial inoculation point. For altered nutrient concentration experiments, the initial concentrations of tryptone and yeast extract were adjusted to be 0.05, 0.1, 0.5, 0.75, or 2.0 times of the regular nutrient concentration, which is referred to as a concentration of 1. To evaluate the effect of phage number, the initial phage stock was serially diluted ten-fold and then spotted on the plate. In experiments conducted with λ phage, the swim plates were supplemented with 10 mM MgSO_4_ and 0.2% maltose.

### Phage titer

Serial dilutions of the phage stock were made and 100 μl of each dilution was added to host bacterial cells with an OD_600_ of 1.0. Bacteria-phage mixtures were incubated for 10 min at room temperature. Each mix received 4 ml of pre-heated 0.5% soft agar and was then overlaid on LB plates. Plates were incubated at 37°C for 4–6 h. The titer of the phage stock was determined by counting the plaques on the plate that yielded between 20 to 200 plaque forming units and multiplying the number by the dilution factor.

### χ phage preparation

Dilutions of phage suspensions mixed with bacteria were plated to achieve confluent lysis as described in the phage titer protocol using 0.35% agar for the overlay. Following formation of plaques, 5 ml of TM buffer (20 mM Tris/HCl [pH = 7.5], 10 mM MgSO_4_) was added to each plate and incubated on a shaking platform at 4°C for a minimum of 6 h. The soft agar/buffer mixture was collected, pooled, and bacteria were lysed by adding chloroform to at final concentration of 0.02%. Samples were mixed vigorously for 1 min, transferred to glass tubes, and centrifuged at 10,000 x g for 15 min at room temperature. The supernatant was passed through a 0.45 μm filter and NaCl was added to a final concentration of 4%. The protocol of phage preparation was followed as described in [60]. The final phage stock was stored in TM buffer at 4°C.

### Bacteria and phage quantifications

Phage drop assays were conducted as described above. At the 14-hour end point, different areas of the plate were sampled by taking agar plugs using a 10 ml syringe barrel with plunger. Each agar plug was placed in 1 ml of 0.85% saline and incubated at room temperature for 10 min with shaking to allow even mixture of the agar. Serial dilutions of each sample were plated on LB agar plates and incubated at 37°C overnight. For phage quantifications, 100 μl of chloroform was added to each sample. The number of phage particles present in each sample was quantified as described in the phage titer protocol. Densities reported correspond to plaque forming units (for phage) or colony forming units (for bacteria). To compare with model results, the volume densities were converted to area densities, based on 0.5 cm thickness in the agar, i.e., area density (cm^-2^) = volume density (CFU/cm^3^ or PFU/cm^3^) × 0.5 cm.

### Model setup

We constructed a mean-field diffusion-drift-reaction model for the bacteria-phage system. Our model includes four variables: density of susceptible bacteria *B*(*x*,*t*), density of infected bacteria *L*(*x*,*t*), density of phages *P*(*x*,*t*), and nutrient concentration *n*(*x*,*t*). The motility and chemotaxis of bacteria are represented by the Keller-Segel type diffusion and advective terms widely used in the literature [61, 62]. The equations governing the spatiotemporal dynamics of bacteria, phages and nutrient read as Eqs (1) ~ (4).

Susceptible bacteria:

$$\begin{array}{l} \frac{\partial B}{\partial t}=\underset{cell\;diffusion}{\underbrace{D_{Bmax}\nabla [{\left(\frac{n}{{n+K}_{v}}\right)}^{2}\nabla B]}}-\underset{cell\;drift}{\underbrace{D_{Bmax}\alpha_{c}\nabla [{\left(\frac{n}{{n+K}_{v}}\right)}^{2}\frac{K_{c}}{{\left({n+K}_{c}\right)}^{2}}B\nabla n]}}\\ \;-\underset{phage\;adsorption}{\underbrace{\eta \frac{K_{b}}{B+L+K_{b}}BP}}+\underset{cell\;division}{\underbrace{g_{max}\frac{n}{n+K_{n}}B}} \end{array}$$ (1)

Infected bacteria:

$$\begin{array}{l} \frac{\partial L}{\partial t}=\underset{cell\;diffusion}{\underbrace{D_{Bmax}\nabla [{\left(\frac{n}{{n+K}_{v}}\right)}^{2}\nabla L]}}-\underset{cell\;drift}{\underbrace{D_{Bmax}\alpha_{c}\nabla [{\left(\frac{n}{{n+K}_{v}}\right)}^{2}\frac{K_{c}}{{\left({n+K}_{c}\right)}^{2}}L\nabla n]}}\\ \;+\underset{phage\;adsorption}{\underbrace{\eta \frac{K_{b}}{B+L+K_{b}}BP}}-\underset{cell\;lysis}{\underbrace{k_{l}Ln}} \end{array}$$ (2)

Phages:

$$\frac{\partial P}{\partial t}=\underset{phage\;diffusion}{\underbrace{D_{P}\nabla^{2}P}}-\underset{phage\;multi-adsorption}{\underbrace{\eta \frac{K_{b}}{B+L+K_{b}}\left(L+B\right)P}}+\underset{phage\;bursting}{\underbrace{\beta k_{l}Ln}}$$ (3)

Nutrient:

$$\frac{\partial n}{\partial t}=\underset{nutrient\;diffusion}{\underbrace{D_{n}\nabla^{2}n}}-\underset{nutrient\;consumption\;by\;bacteria}{\underbrace{\lambda g_{max}\frac{n}{n+K_{n}}(B+L)}}$$ (4)

Eqs (1) ~ (4) incorporate the following model assumptions.

1. The division rate of susceptible bacteria follows the Monod rate law [63].
2. Division of the infected bacteria is neglected because they are likely lysed before dividing. But they consume nutrients at the same rate as susceptible bacteria (changing this rate does not affect the qualitative behavior of the model).
3. The phage adsorption rate decreases with increasing bacterial density. This is how New Assumption (ii) in Results is implemented.
4. Multi-adsorption is considered, i.e., phages can be adsorbed onto bacteria that are already infected.
5. Because phage assembly requires energy, we assume that the lysis period elongates as nutrient level decreases. This is how New Assumption (i) in Results is implemented.
6. Because bacterial motility requires energy, it depends on nutrient level. This dependence is reflected by the fraction containing *K*_*v*_ in both the diffusion and chemotaxis terms. This applies to both susceptible and infected bacteria.

The variables and parameters of the model are summarized in Table 2.

**Table 2:** Parameters of mathematical model.

Symbols	Meaning	Default values	Sources
*D*_*Bmax*_	Maximum diffusion coefficient of bacterial population (effective diffusion coefficient when bacteria assume maximum motility)	5.25×10^5^ μm^2^h^-1^	Estimated from cell velocity and tumbling frequency [43, 64]
*K*_*v*_	Nutrient level for half maximum cell motility	0.001 μm^-2^	Fitting to experimental data *
*K*_*c*_	Nutrient level for half maximum chemotactic efficiency	0.02 μm^-2^	Fitting to experimental data *
*α*_*c*_	Chemotactic efficiency	2	Fitting to experimental data *
*D*_*P*_	Diffusion coefficient of phage particles	1 μm^2^h^-1^	Particle size ~ 0.1 or 0.05 μm; diffusion coefficient ~ 1/particle size
*D*_*n*_	Diffusion coefficient of nutrient	4.5×10^6^ μm^2^h^-1^	[65]
*η*	Phage adsorption rate constant	8×10^4^ μm^2^h^-1^	[66, 67]
*β*	Phage burst size	80	[68]
*k*_*l*_	Lysis rate constant of infected bacteria (~ 1 / latency period)	2 μm^2^h^-1^	[67, 69]
*g*_*max*_	Maximum division rate of bacteria	6 h^-1^	Fitting to experimental data *
*K*_*n*_	Half-saturation nutrient concentration for Monod growth law	0.1 μm^-2^	Fitting to experimental data *
*λ*	Bacterial growth yield (ratio between quantity of produced bacteria and quantity of consumed nutrient)	0.2	Fitting to experimental data *
*K*_*b*_	Bacteria density for half maximum phage adsorption rate	0.1 μm^-2^	Fitting to experimental sector-shaped lysis pattern
*B*_0_	Number of inoculated bacteria	1×10^7^	Experimental setup
*P*_0_	Number of inoculated phages	1.5×10^8^	Experimental setup
*n*_0_	Initial area density of nutrient	1 μm^-2^	Normalized (*K*_c_, *K*_n_ and *λ* scale with *n*_0_)
*r*	Radius of inoculation circle of bacteria and phage	0.25 cm	Experimental setup
*R*	Radius of plate	5.5 cm	Experimental setup

* Fitting to experimentally observed expansion rate of bacterial swim ring.

## Supporting information

S1 Fig Both direct and indirect negative dependences of phage proliferation on bacterial density are necessary for generating straight radial boundaries in the lysis pattern. (a) Simulated lysis pattern formation with both Assumptions (i) and (ii). Same results as Fig 1B, second row. (b) Simulated lysis pattern formation without Assumption (i), but with Assumption (ii). (c) Simulated lysis pattern formation without Assumption (ii), but with Assumption (i). (d) Simulated lysis pattern formation without both Assumptions. As described in Results, Assumption (i) states that nutrient deficiency inhibits phage replication, and Assumption (ii) states that high bacterial density inhibits phage production.
(TIF)

S2 Fig Model results show steady bacteria and phage densities at the expanding front after the phage initiation zone emerges. (a) Lysis patterns over time. Blue dashed line: cutline over which the density profiles are plotted in (b). (b) Density profiles of susceptible bacteria over the cutline at the labeled times. (c) Density profiles of phages over the cutline at the labeled times.
(TIF)

S3 Fig Simulation results with various numbers of inoculated bacteria. (TIF)

S4 Fig Lysis pattern with straight radial boundaries requires balance between intrinsic properties of bacteria and phage. Simulated lysis patterns with (a) various phage adsorption rate constants and bacterial diffusion coefficients, (b) various bacterial division rate constants and bacterial diffusion coefficients, (c) various phage adsorption rate constants and chemotactic efficiencies, and (d) various bacterial division rate constants and chemotactic efficiencies.
(TIF)

S5 Fig Experimental results of *S*. Typhimurium strains lacking chemoreceptors. (TIF)

S6 Fig Lysis patterns with different bacterial motility. (a) Experimental results with different agar densities. Plates were incubated until bacterial swim rings reached the edge of the plate (7 h for 0.2%, 9 h for 0.25%, and 14 h for 0.3% agar), and the images were taken at the end of the experiments. (b) Simulated lysis patterns with various bacterial diffusion coefficients and nutrient diffusion efficiencies. The bacterial diffusion coefficient in the model reflects the efficiency of bacterial motility.
(TIF)

S7 Fig Co-propagation between phage and bacteria is reflected by the straight radial boundary of lysis pattern. (a) Lysis patterns and (b) corresponding spatial patterns of phages with various bacterial diffusion coefficients and phage adsorption rate constants. Superposition of (a) and (b) gives Fig 5A in the main text. Grey shadowed staircase: potential trajectory of evolutionary arms race on which phages and bacteria maintain balance with each other.
(TIF)

S8 Fig Effect of temperature on lysis pattern. *Salmonella enterica* serovar Typhimurium 14028s was incubated with χ phage at 37˚C (temperature used for all other experiments except where otherwise indicated), 30˚C, or room temperature (RT). Plates were incubated until the bacterial swim rings reached the edge of the plate (14 h at 37˚C, 22.5 h at 30˚C, 38 h at RT). Although there are slight differences in the lysis angle, the overall shape of the pattern remains largely the same.
(TIF)

S9 Fig The shape of lysis area depends on the competition between phages and bacteria, but not the initial nutrient level. Simulated lysis patterns with various initial nutrient levels and (a) bacterial division rate constants, (b) phage adsorption rate constants, (c) bacterial diffusion coefficients, (d) chemotactic efficiencies.
(TIF)

S1 Movie Time evolution of the lysis pattern in experiment and model. Each frame displays corresponding time points in experiment vs. model. Total time 14 h. Color bar represents density of bacteria (cm^-2^) in model.
(GIF)
