# Supplementary figures and images for: Formation of phage lysis patterns and implications on co-propagation of phages and motile host bacteria
Source: PLoS Comput Biol. 2020 Mar 13;16(3):e1007236. doi: 10.1371/journal.pcbi.1007236 (PMC7108739; doi:10.1371/journal.pcbi.1007236)

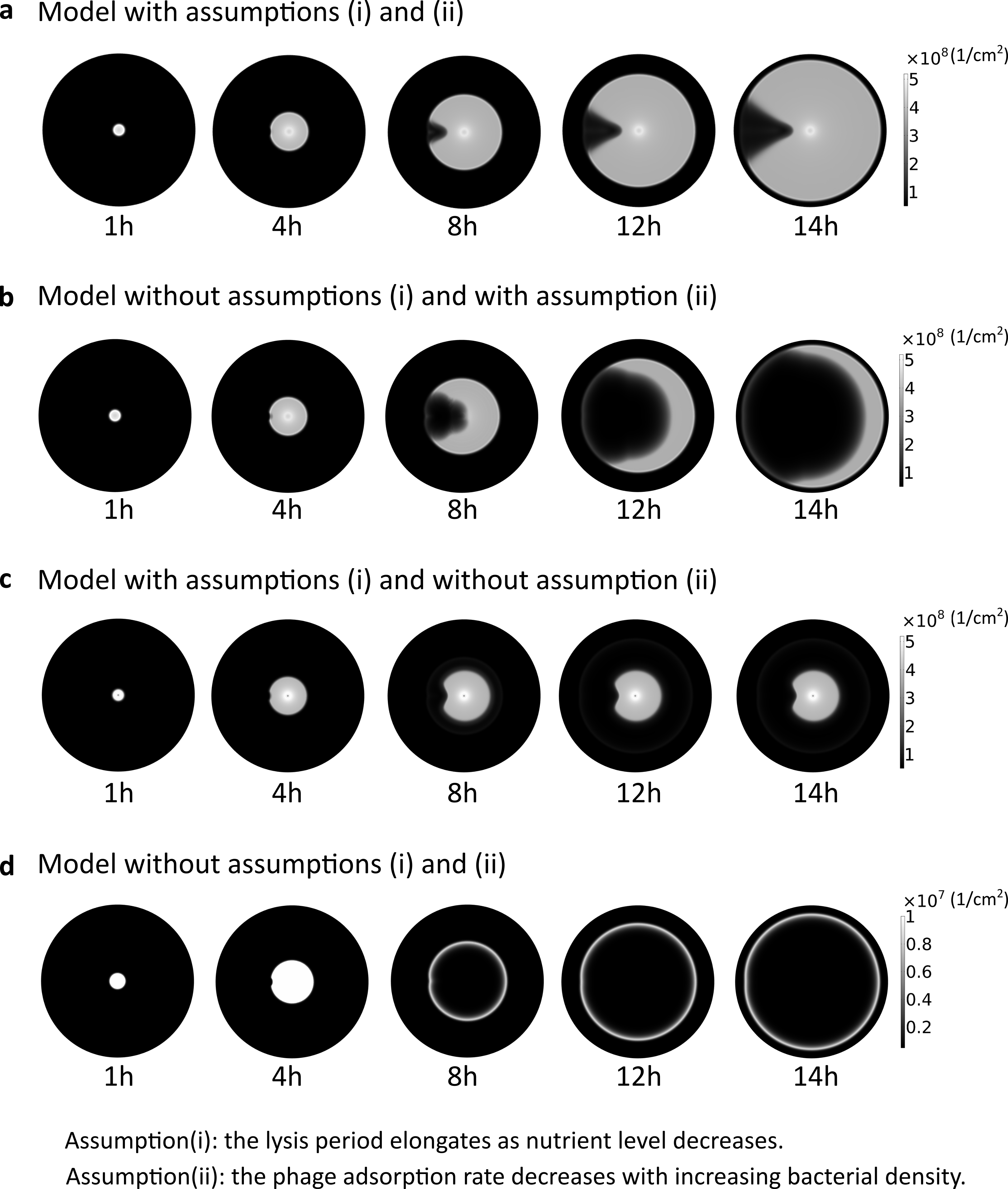

Supplement: S1 Fig — (a) Simulated lysis pattern formation with both Assumptions (i) and (ii). Same results as Fig 1B, second row. (b) Simulated lysis pattern formation without Assumption (i), but with Assumption (ii). (c) Simulated lysis pattern formation without Assumption (ii), but with Assumption (i). (d) Simulated lysis pattern formation without both Assumptions. As described in Results, Assumption (i) states that nutrient deficiency inhibits phage replication, and Assumption (ii) states that high bacterial density inhibits phage production. (TIF) [file pcbi.1007236.s001.tif]

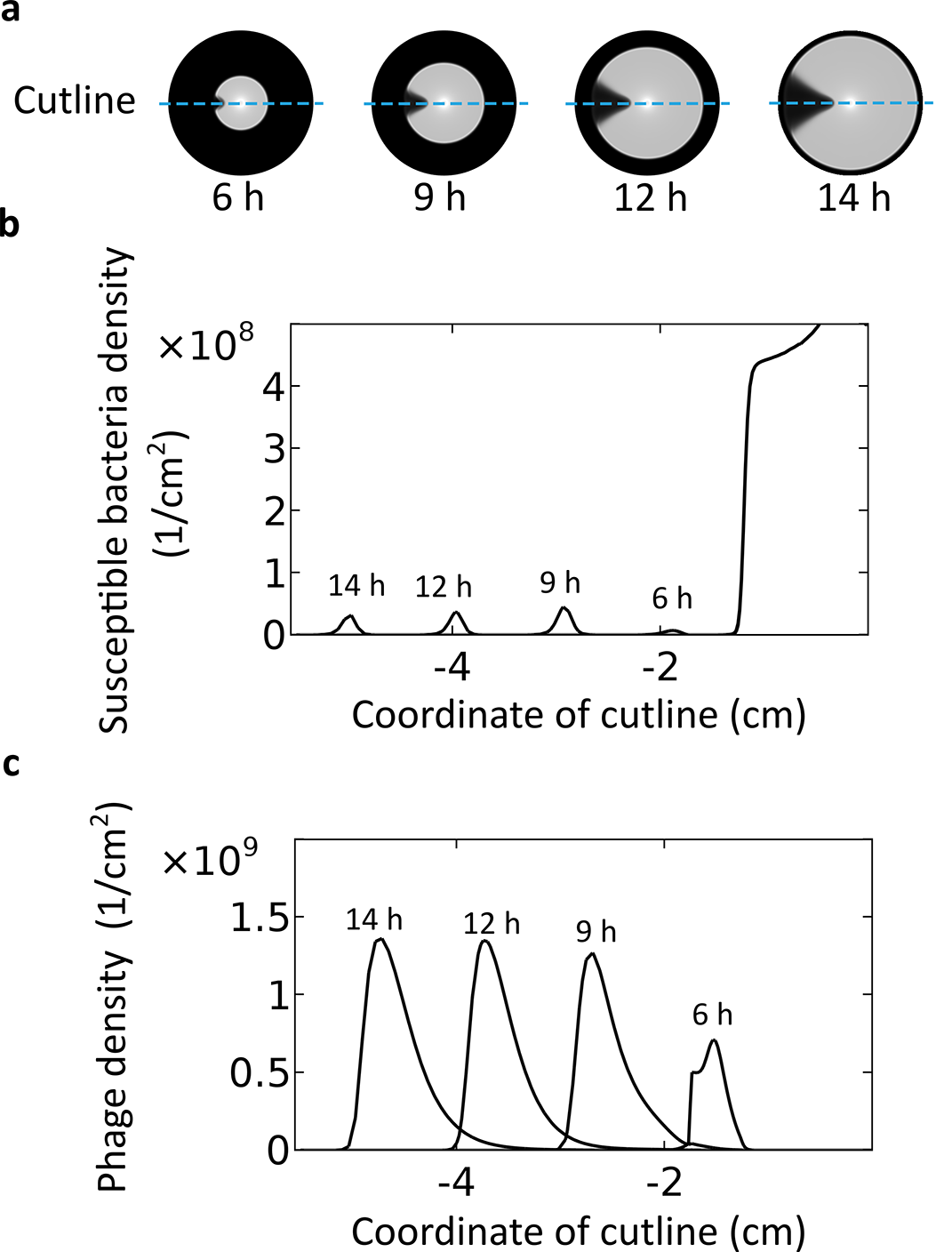

Supplement: S2 Fig — (a) Lysis patterns over time. Blue dashed line: cutline over which the density profiles are plotted in (b). (b) Density profiles of susceptible bacteria over the cutline at the labeled times. (c) Density profiles of phages over the cutline at the labeled times. (TIF) [file pcbi.1007236.s002.tif]

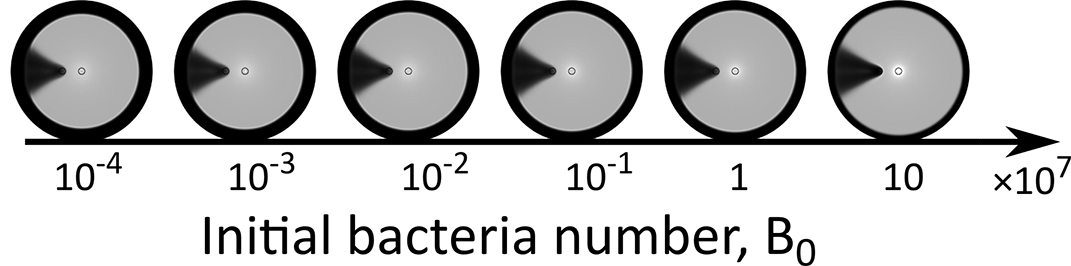

Supplement: S3 Fig — (TIF) [file pcbi.1007236.s003.tif]

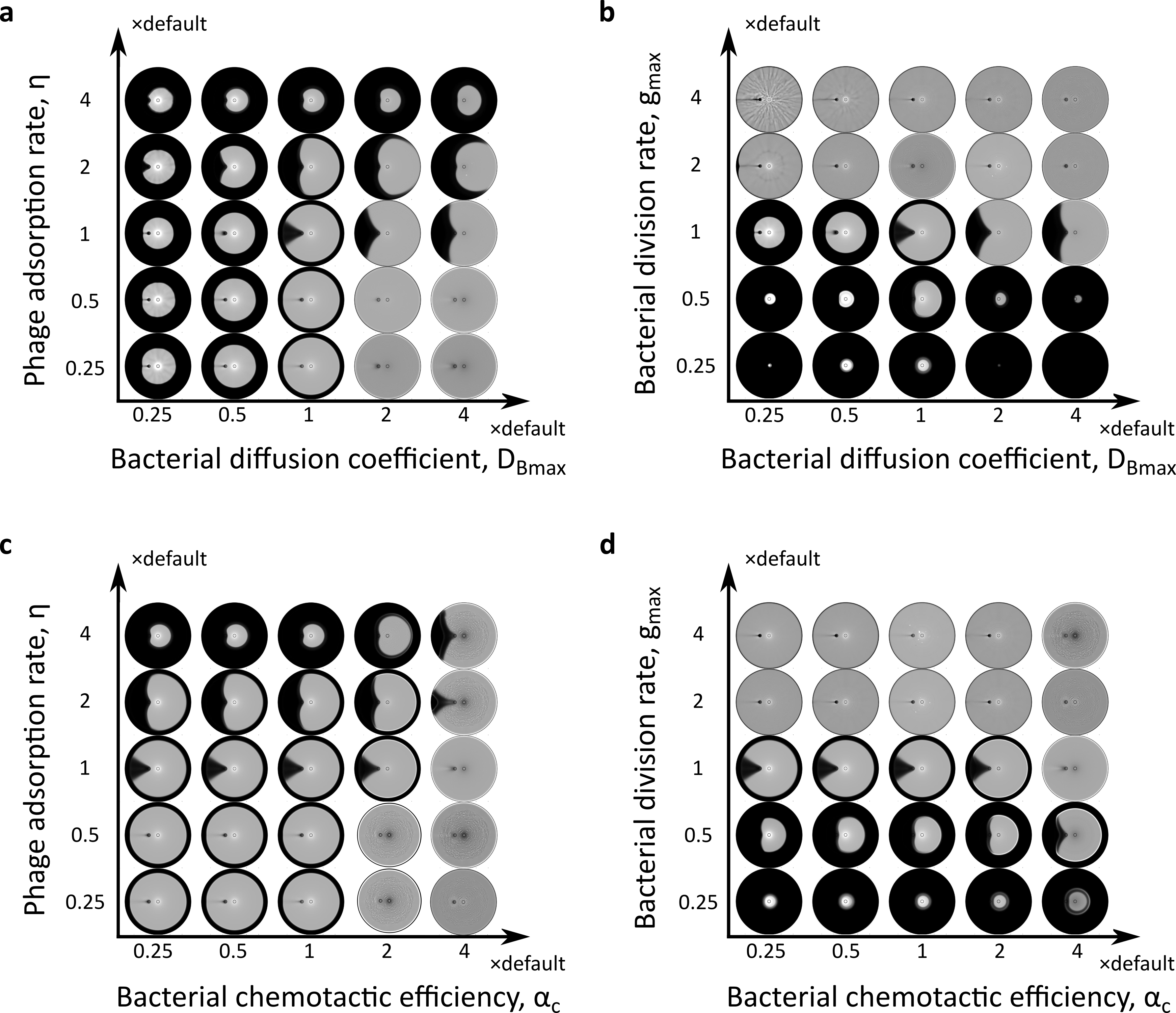

Supplement: S4 Fig — Simulated lysis patterns with (a) various phage adsorption rate constants and bacterial diffusion coefficients, (b) various bacterial division rate constants and bacterial diffusion coefficients, (c) various phage adsorption rate constants and chemotactic efficiencies, and (d) various bacterial division rate constants and chemotactic efficiencies. (TIF) [file pcbi.1007236.s004.tif]

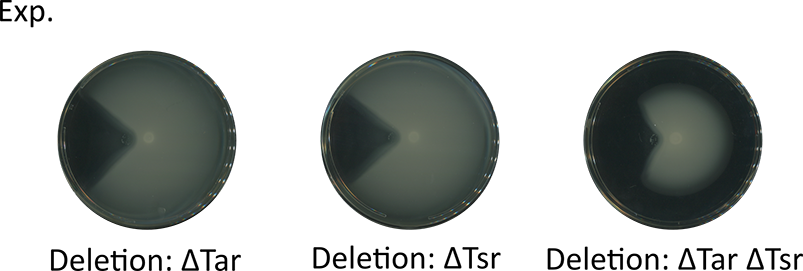

Supplement: S5 Fig — (TIF) [file pcbi.1007236.s005.tif]

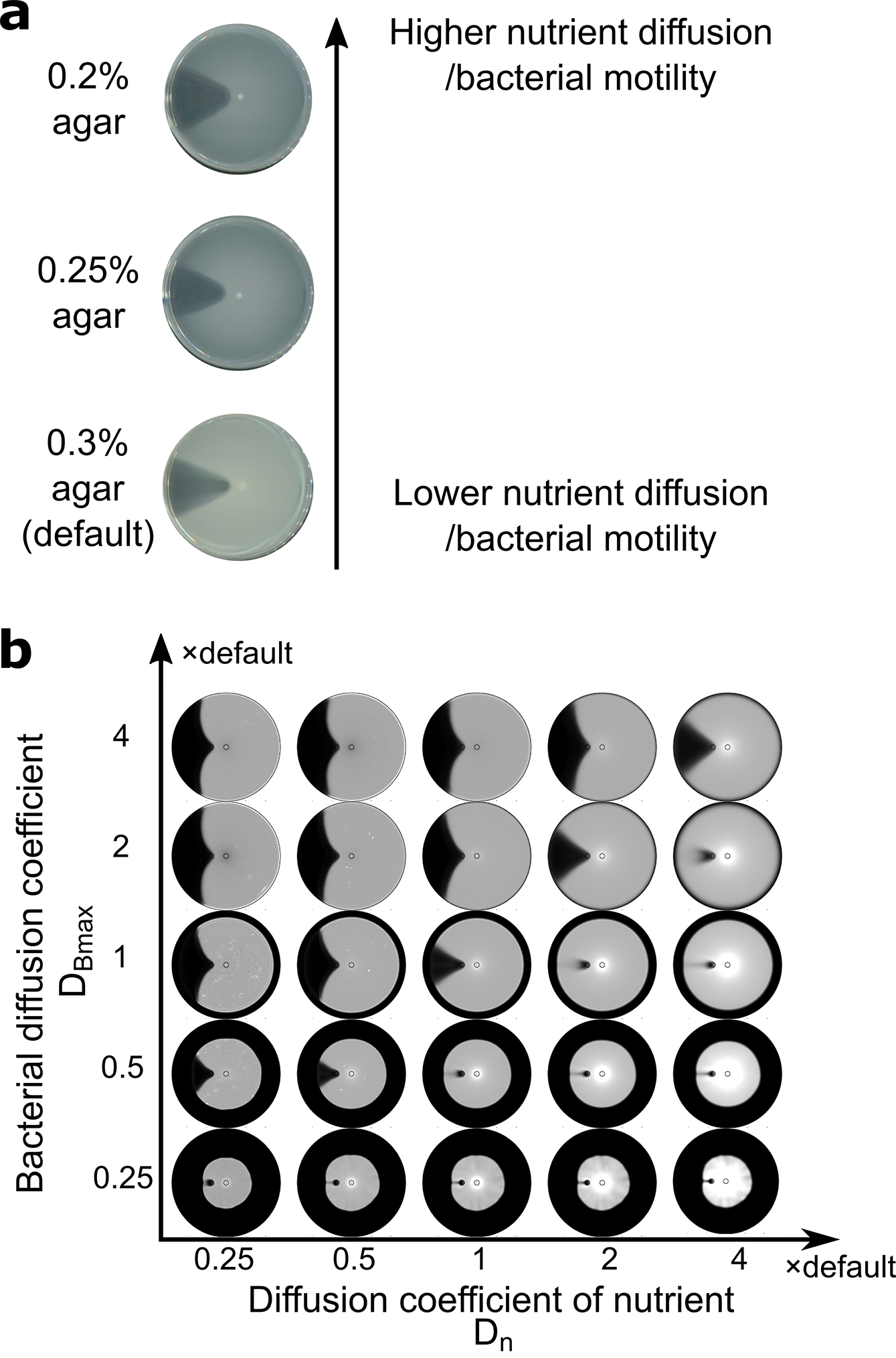

Supplement: S6 Fig — (a) Experimental results with different agar densities. Plates were incubated until bacterial swim rings reached the edge of the plate (7 h for 0.2%, 9 h for 0.25%, and 14 h for 0.3% agar), and the images were taken at the end of the experiments. (b) Simulated lysis patterns with various bacterial diffusion coefficients and nutrient diffusion efficiencies. The bacterial diffusion coefficient in the model reflects the efficiency of bacterial motility. (TIF) [file pcbi.1007236.s006.tif]

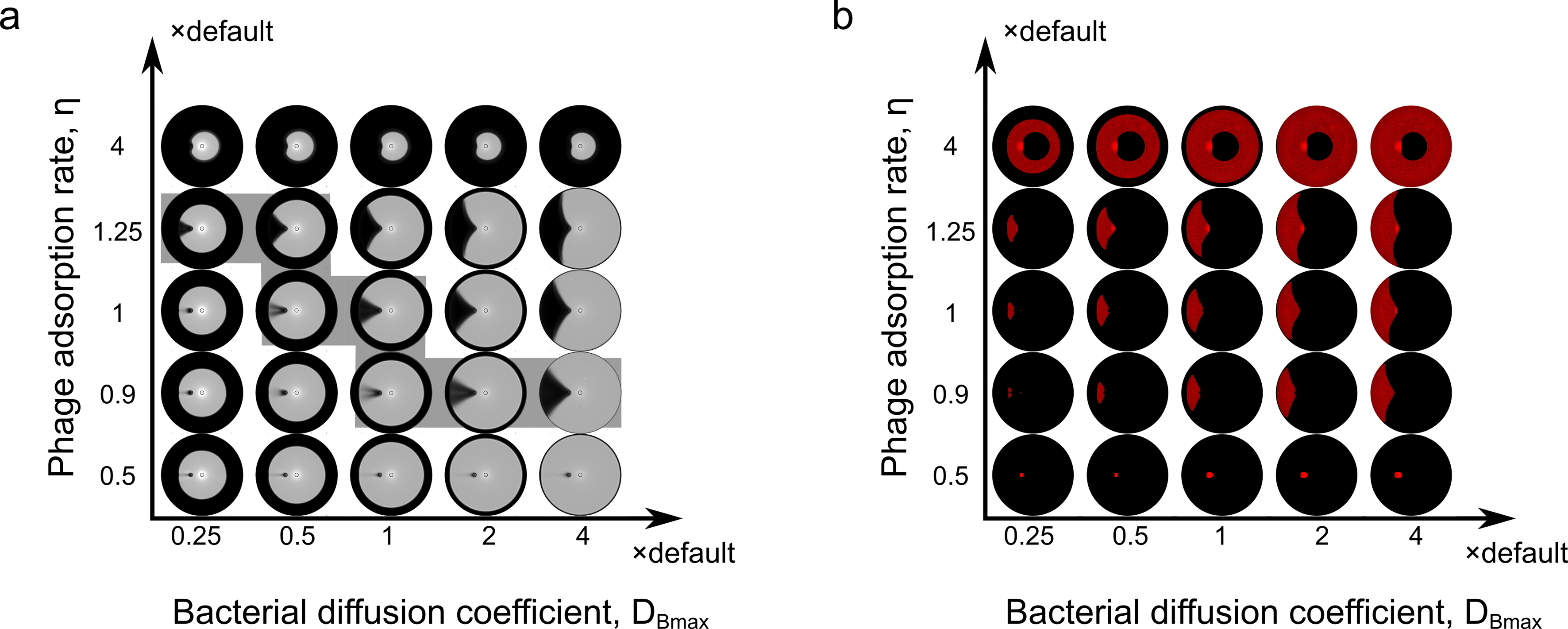

Supplement: S7 Fig — (a) Lysis patterns and (b) corresponding spatial patterns of phages with various bacterial diffusion coefficients and phage adsorption rate constants. Superposition of (a) and (b) gives Fig 5A in the main text. Grey shadowed staircase: potential trajectory of evolutionary arms race on which phages and bacteria maintain balance with each other. (TIF) [file pcbi.1007236.s007.tif]

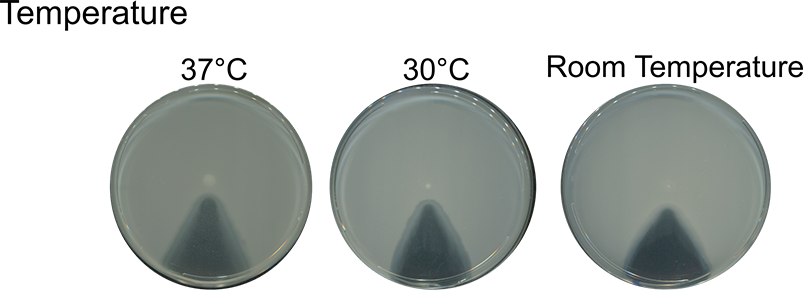

Supplement: S8 Fig — Salmonella enterica serovar Typhimurium 14028s was incubated with χ phage at 37˚C (temperature used for all other experiments except where otherwise indicated), 30˚C, or room temperature (RT). Plates were incubated until the bacterial swim rings reached the edge of the plate (14 h at 37˚C, 22.5 h at 30˚C, 38 h at RT). Although there are slight differences in the lysis angle, the overall shape of the pattern remains largely the same. (TIF) [file pcbi.1007236.s008.tif]

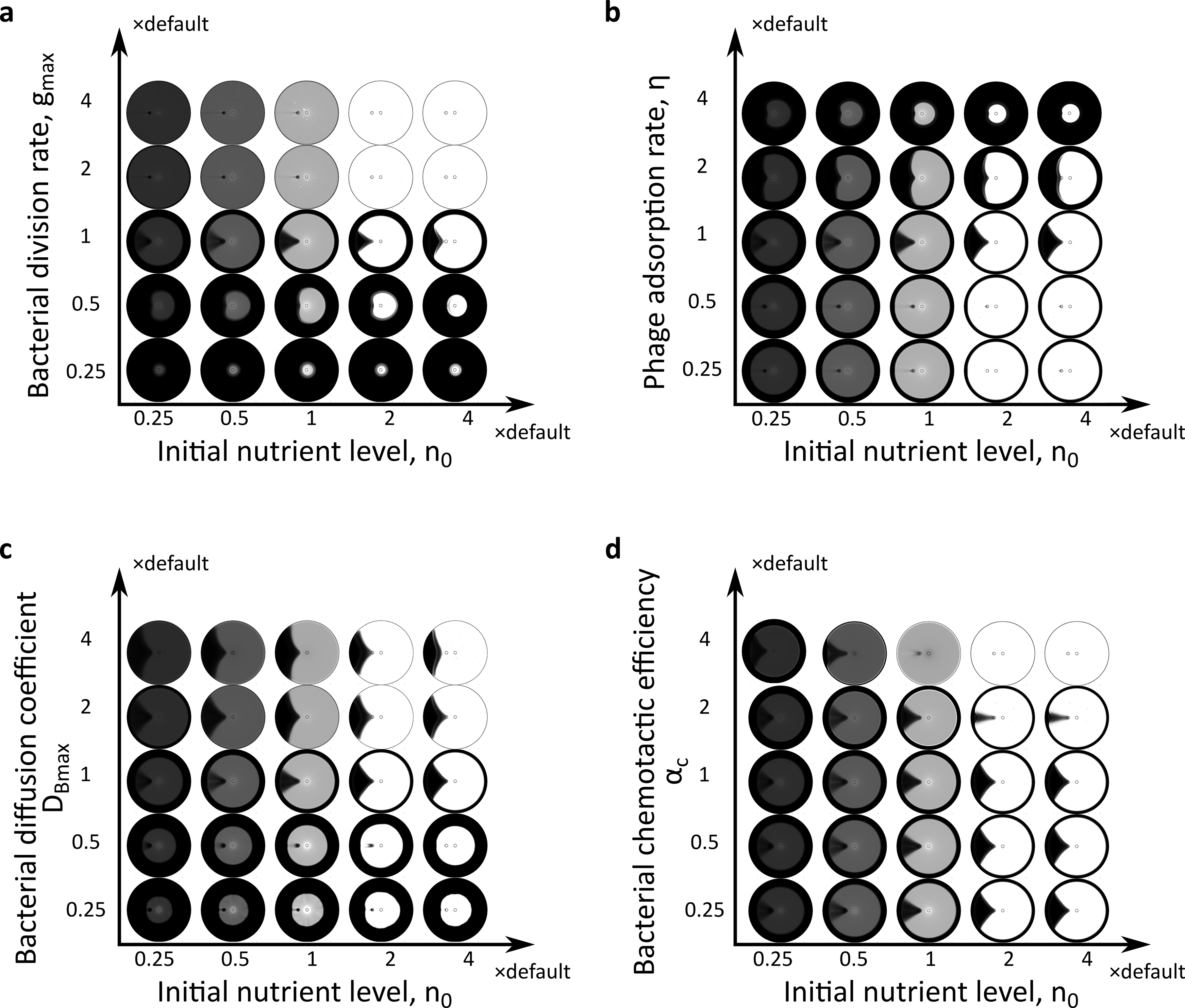

Supplement: S9 Fig — Simulated lysis patterns with various initial nutrient levels and (a) bacterial division rate constants, (b) phage adsorption rate constants, (c) bacterial diffusion coefficients, (d) chemotactic efficiencies. (TIF) [file pcbi.1007236.s009.tif]

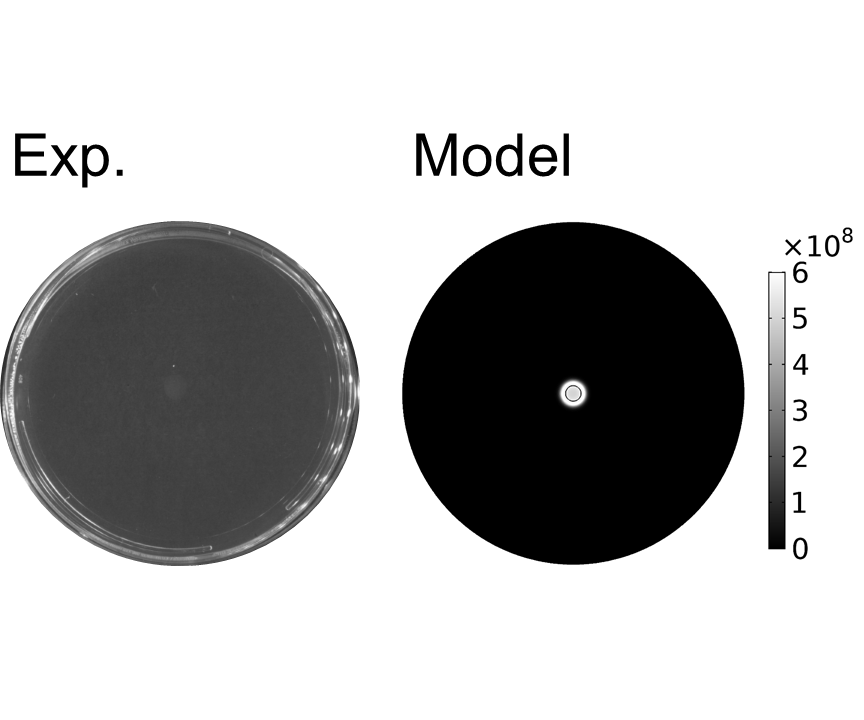

Supplement: S1 Movie — Each frame displays corresponding time points in experiment vs. model. Total time 14 h. Color bar represents density of bacteria (cm-2) in model. (GIF) [file pcbi.1007236.s010.gif]
